# Effect of Vitamin D Supplementation on Primary Dysmenorrhea: A Systematic Review and Meta-Analysis of Randomized Clinical Trials

**DOI:** 10.3390/nu15132830

**Published:** 2023-06-21

**Authors:** Yi-Chun Chen, Yi-Fen Chiang, Ying-Jiun Lin, Ko-Chieh Huang, Hsin-Yuan Chen, Nadia M. Hamdy, Tsui-Chin Huang, Hsin-Yi Chang, Tzong-Ming Shieh, Yun-Ju Huang, Shih-Min Hsia

**Affiliations:** 1 School of Nutrition and Health Sciences, College of Nutrition, Taipei Medical University, Taipei 110301, Taiwan; 2 Department of Family and Community Medicine, Cheng Hsin General Hospital, Taipei 112401, Taiwan; 3 Biochemistry Department, Faculty of Pharmacy, Ain Shams University, Abassia, Cairo 11566, Egypt; 4 Graduate Institute of Cancer Biology and Drug Discovery, College of Medical Science and Technology, Taipei Medical University, Taipei 110301, Taiwan; 5 Graduate Institute of Medical Science, National Defense Medical Center, Taipei 114201, Taiwan; 6 School of Dentistry, College of Dentistry, China Medical University, Taichung 40402, Taiwan; 7 Department of Biotechnology and Food Technology, Southern Taiwan University of Science and Technology, Tainan City 710301, Taiwan; 8 School of Food and Safety, Taipei Medical University, Taipei 110301, Taiwan; 9 Nutrition Research Center, Taipei Medical University Hospital, Taipei 110301, Taiwan; 10 Graduate Institute of Metabolism and Obesity Sciences, College of Nutrition, Taipei Medical University, Taipei 110301, Taiwan; 11 TMU Research Center for Digestive Medicine, Taipei Medical University, Taipei 110301, Taiwan

**Keywords:** vitamin D, dysmenorrhea, systemic review meta-analysis (SRMA), randomized controlled trials (RCTs), pain relief, pain degree, Cochrane risk-of-bias (RoB 2)

## Abstract

Dysmenorrhea causes pain and inconvenience during menstruation. In addition to medication, natural compounds are widely used to relieve various types of pain. In this study, we aimed to assess the effects of vitamin D (vit. D) supplementation in relieving the symptoms of primary dysmenorrhea. A comprehensive systematic database search of randomized controlled trials (RCTs) was performed. Oral forms of vit. D supplementation were included and compared with a placebo or standard care. The degree of dysmenorrhea pain was measured with a visual analogue scale or numerical rating scale. Outcomes were compared using the standardized mean difference (SMD) and 95% confidence intervals (CIs) in a meta-analysis. RCTs were assessed using the Cochrane risk-of-bias v2 (RoB 2) tool. The meta-analysis included 8 randomized controlled trials involving 695 participants. The results of the quantitative analysis showed a significantly lower degree of pain in the vit. D versus placebo in those with dysmenorrhea (SMD: −1.404, 95% CI: −2.078 to −0.731). The results of subgroup analysis revealed that pain lessened when the average weekly dose of vit. D was over 50,000 IU, in which dysmenorrhea was relieved regardless of whether vit. D was administered for more or less than 70 days and in any dose interval. The results revealed that vit. D treatment substantially reduced the pain level in the primary dysmenorrhea population. We concluded that vit. D supplementation is an alternative treatment for relieving the pain symptoms of dysmenorrhea.

## 1. Introduction

Based on pathophysiology, dysmenorrhea/menstrual distress is categorized into either primary or secondary dysmenorrhea. According to the NHGRI-EBI Catalog of human genome-wide association studies (GWASs; https://www.ebi.ac.uk/gwas/efotraits/HP_0100607; accessed on 8 March 2023), eight triads have been reported: discomfort and pain occurring only during menstrual bleeding (UKB data field 21026); discomfort and pain occurring only during menstrual bleeding (UKB data field 21026; gene-based burden); dysmenorrhea (PheCode 626.2); ICD10 N92.6: irregular menstruation, unspecified; ICD10 N92.6: irregular menstruation, unspecified (gene-based burden); ICD10 N94.6: dysmenorrhea, unspecified; ICD10 N94.6: Dysmenorrhea, unspecified (gene-based burden), and primary dysmenorrhea.

The painful symptoms of primary dysmenorrhea are often described as spasmodic cramping in the lower abdomen, sometimes extending to the lower back or thighs [1,2]. These painful symptoms appear just before and/or during menstrual periods in those with normal pelvic anatomy, usually beginning 6–24 months after menarche in their adolescence [1,2]. Secondary dysmenorrhea, in contrast, usually begins 2 years after menarche but can occur any time during puberty. The symptoms of secondary dysmenorrhea may result from various anatomical pathologies, including endometriosis, adenomyosis, fibroids, and pelvic inflammatory disease(s) [3].

The presence of dysmenorrheal symptoms substantially lowers the quality of life in comparison with those who do not experience these symptoms [4]. The degree of painful menstrual cramps was reported to be as intense as renal colic pain [5]. Moreover, various aspects of life are affected, including physical and social functions, imposing limitations due to emotional or physical problems/hindrances, body pain, decreased vitality, mental health effects, and a decreased general perception of health [6].

The current standard treatment for dysmenorrhea includes nonsteroidal anti-inflammatory drugs (NSAIDs) and oral contraceptive pills (OCPs). However, the adverse effects of NSAIDs include peptic ulcers, hepatic and renal disorders, and allergic reactions [7]. Additionally, OCPs reduce the levels of PGs but they suppress ovulation, increasing the tendency of blood hypercoagulation, suspected breast-related disease(s), pregnancy problems, and cultural and religious conflicts/concerns [8]. Promising nondrug measures for relieving symptoms include regular exercise; the local application of a heating pad; consumption of ginger, cinnamon, curcumin, fish oil, vitamins, and minerals; acupuncture; or even surgery [1,9].

Vitamin D/vitamin D3/cholecalciferol/calciol (KEGG COMPOUND_C05443 https://www.genome.jp/entry/C05443 (accessed on 6 March 2023)) [10] was retrieved from the Kyoto Encyclopedia of Genes and Genomes (KEGG). Vit. D, per its anti-inflammatory role, may be another feasible treatment option for dysmenorrhea. Additionally, the involvement of the vit. D receptor (VDR) gene in menstrual dysfunction pathogenesis [11], supports vit. D as a feasible treatment for the alleviation of dysmenorrhea symptoms. Additionally, muscle hypercontraction is related to calcium influx [12]; calcium, as a stabilizing agent, could regulate nerve signals and the related dysmenorrhea symptoms [9]. Vit. D, also known as calciferol, can modulate the levels of PG and calcium homeostasis, so it may alleviate the symptoms of dysmenorrhea [13].

The two common forms of vit. D supplements are ergocalciferol (vit. D2; derived from plants) and cholecalciferol (vit. D3; derived from animals). Most reports have stated that vit. D3 is more efficiently active than vit. D2 in increasing serum 25[OH]D levels, which is a stable and commonly used marker for monitoring the status of body vit. D levels [14].

Due to the side effects or contraindications of conventional NSAIDs and OCPs, approximately 15% of dysmenorrhea-affected women drop out of or withdraw from using these medications [15]. Therefore, finding potential therapeutic options that can improve the quality of life of these people is worth exploring. As such, in this SRMA, we aimed to examine the effectiveness of the use of vit. D for the relief of primary dysmenorrheal pain as a treatment option instead of NSAIDs or OCPs. Moreover, we aimed to explore whether the effect of vit. D intervention would differ between regimens.

## 2. Materials and Methods

### 2.1. Study Design

The systematic review protocol was PROSPERO registered with the following ID number: CRD42023407771.

### 2.2. Search Strategy

A literature search was conducted in PubMed, Medline, Embase, Cochrane Library, Web of Science, Scopus, and Cinahl to explore the effectiveness of vit. D treatment in alleviating primary dysmenorrheal pain. The keywords/search terms/(MeSH) used for the search were as follows: “dysmenorrhea” OR “dysmenorrhoea“ OR “primary dysmenorrhea” OR “primary dysmenorrhoea” OR “menstrual pain” OR “painful menstruation” OR “menstrual cramps” AND “cholecalciferol” OR “hydroxycholecalciferols” OR “vitamin D” OR “vitamin D3” OR “25-hydroxyvitamin D3” OR “25(OH)D”. Detailed dysmenorrhea MeSH–NCBI can be retrieved from https://www.ncbi.nlm.nih.gov/mesh/?term=dysmenorrhea (accessed on 6 March 2023).

### 2.3. Inclusion and Exclusion Criteria; PICO

The inclusion criteria were as follows: English language only in all fields; search was restricted to 10 years from January 2012 to March 2023; involving randomized clinical trials (RCTs). The conditions for inclusion in the study included the person’s menstrual cycle occurring every 21 to 35 days, with the menstrual period lasting between 3 and 7 days.

The population (P) included women experiencing menstrual distress/pain with no underlying cause (primary dysmenorrhea) and no history of uterine diseases (fibroids, duodenal ulcers, polyps, endometrial hypertrophy, or endometriosis) or ovarian diseases (ovarian cysts or polycystic ovaries); and a normal pelvic examination. The patients had to be in good health and were not receiving any medical treatment including calcium- or magnesium-containing drugs or OCPs.

Intervention studies involving animals or humans and other studies that required ethical approval needed to list the authority that provided the approval and the corresponding ethical approval code, code number, and date of approval.

The exclusion criteria included women who had been diagnosed with secondary dysmenorrhea and other reproductive diseases (uterine fibroids or endometriosis); were using hormonal therapy, OCPs, or using intrauterine devices; if pregnant; those taking other vitamins or minerals; and those not in pain. Reviews, conference abstracts, letters-to-editors, commentaries or editorials, book chapters, case studies, and studies published in languages other than English and studies reporting malignancies were all excluded. Studies with duplicated or without sufficient data were excluded as well.

The comparators (C) were the placebo group. The primary outcome (O) was the degree of primary dysmenorrheal pain relief compared with after vit. D treatment (intervention (I)).

### 2.4. Data Extraction, Synthesis, and Analysis

Data were collected from the previously mentioned databases in March 2023. 

The following data were gathered from each included eligible article: study design, name of the first author, year of publication, country, sample size, definition of response, and results. 

Data were extracted from the visual analogue scale (VAS) evaluated during menstrual cycles.

### 2.5. Quality Assessment and Risk of Bias (ROB)

Between-trial heterogeneity was determined by using chi-square tests; values > 50% were regarded as considerable heterogeneity. Random-effects model was applied to pool individual SMDs. The SMD value indicated vit. D to be a favorable treatment option. Funnel plots and Egger’s test were used to examine potential publication bias. RCTs were assessed using version 2 of the Cochrane risk-of-bias (RoB 2) tool (https://methods.cochrane.org/bias/resources/rob-2-revised-cochrane-risk-bias-tool-randomized-trials (accessed on 16 May 2023)) and the revised tool https://sites.google.com/site/riskofbiastool/welcome/rob-2-0-tool?authuser=0 (accessed on 16 May 2023). 

Two investigators independently assessed the risk of bias and the quality of individual eligible studies and disagreements between the two investigators were resolved by consulting a third investigator.

### 2.6. Statistical Analysis

Data were analyzed by one investigator and confirmed by another, and the data were further revised by a third investigator in the case any discrepancy arose. Vit. D blood level is presented as the standardized mean differences (SMDs). A visual analogue scale (VAS; 0–10) was used as the tool for assessing pain; otherwise, a numerical rating scale (NRS; 0–10) was used. RoB2 is presented as low risk (+), high risk (−), or unknown (?). The I^2^ results revealed a high heterogeneity among the included studies. The overall effect size with 95% confidence intervals (CIs) was used to estimate the association. The heterogeneity between studies was assessed using *I*^2^ (with chi square (Chi^2^**/***x*^2^) among the included studies and interpreted following the guidelines outlined in the *Cochrane Handbook for Systematic Reviews of Interventions* (https://training.cochrane.org/handbook (accessed on 16 May 2023)). Forest plot intercept was done as ROB estimation of the interventions effect on the outcome. Linear regression of the intervention effect Funnel:Egger’s test plot performed for confirmation. The possible explanations for heterogeneity were investigated using random-effects and subgroup analyses. A comprehensive meta-analysis was conducted using comprehensive meta-analysis (CMA) software version 3.0. We used a random-effects model for meta-analysis with the significance set to *p*-values of less than 0.05.

## 3. Results

### 3.1. Eligible Studies Flow Chart

**Figure 1:**
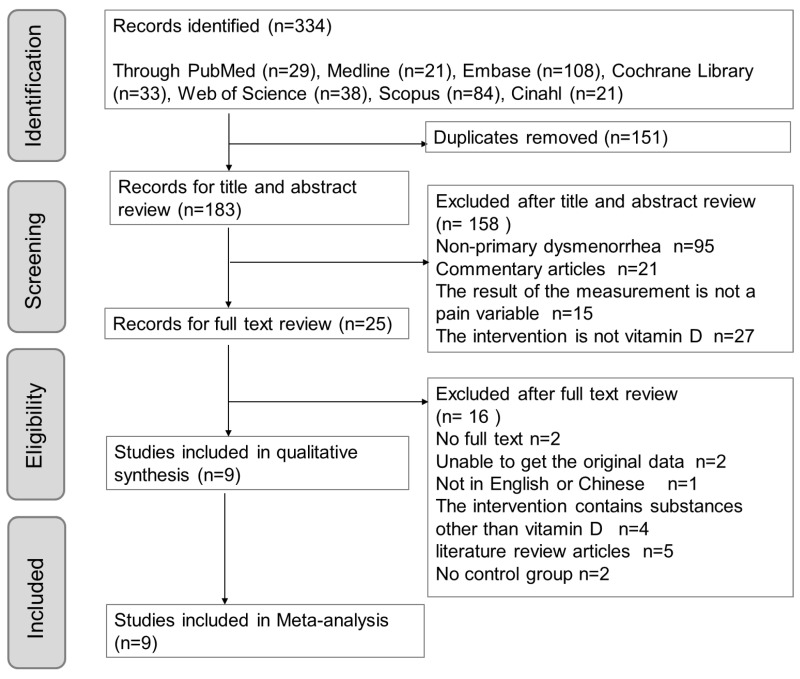
PRISMA flow chart outlining literature search screening and selection procedure.

According to the inclusion and exclusion criteria in this study, we searched seven English databases, including PubMed, Embase, Medline, Cochrane Library, Scopus, Web of Science, and Cinahl. The initial search yielded 334 results using the search strategy and previously mentioned keywords. Among the 183 articles remaining after removing the duplicates, 95 articles focused on nonprimary dysmenorrhea, 21 articles were review articles, 15 articles used non-pain variables for measurement, and 27 studies did not use vit. D as an intervention (used other vitamins). A total of 25 articles were then subjected to a full-text review, of which two articles did not have full text, the raw data of two articles were not available, one article was not in English (Chinese), four articles considered substances other than vit. D in the intervention, and five articles were systematic literature reviews (Figure 1). Finally, nine RCTs were included in the meta-analysis. 

These nine studies that reported the outcomes of interest were included in the meta-analysis for data extraction and statistical analysis. The systematic review and meta-analysis were designed according to and followed PRISMA and meta-analysis guidelines.

### 3.2. Quality ROB

We used the Cochrane Collaboration tool for assessing RoB in RCTs to evaluate and analyze studies quality; with results are shown in Table 1. In terms of selection bias, seven out of nine articles were rated as low risk, whereas two out of nine articles were rated as unknown. These ratings were assigned after explaining the method of random allocation.

Regarding performance bias, seven out of nine articles rated as low risk. In these studies, where subjects were blinded and unaware of which group, they were assigned to, thereby reducing the psychological impact. However, one out of nine articles were rated as high risk because subjects in that particular group were aware of the intervention or control/placebo to which they were allocated. Additionally, one out of the nine articles were rated as having an unknown risk, because authors did not state whether blinding was performed or not.

Regarding detection bias, six out of the nine studies were rated as low risk, and the article mentioned the blinding of the prognostic evaluator or that blinding would not have affected results judgment. Two studies (2/9) were assessed as high risk, because the prognostic evaluators did not use blinding, which could have affected results during the study. In one study, authors used questionnaire as the measurement tool and per, they did not mention whether the examiners were blinded, so they were rated as unknown risks.

In terms of attrition bias, the nine articles were rated as low risk with an article attrition rate less than 10%, with the reason for loss-of-cases clearly explained in the article.

Regarding reporting bias, all articles were consistent with the SRMA direction and observation goals, which were clear and complete and were assessed as low risk/low RoB.

### 3.3. RCT Characteristics

Among the nine included articles, eight were published in the last 10 years (2013 to 2023). The study population included young people aged 13–40 years from Iran, India, and Saudi Arabia. The total sample size at baseline was 695. The intervals of menstrual cycles were 21 to 35 days. A visual analogue scale (VAS; 0–10) was used as the tool to assess pain in eight out of the nine studies. A numerical rating scale (NRS; 0–10) was used in one study (1/9). The study duration ranged from 2 to 4 months. Of the nine studies, six were designed as double-blind RCTs, and the remaining studies included two single-blind RCTs, and one was an open-label trial (Table 2).

### 3.4. Intervention Details and Outcome Measurements

Table 3 provides the intervention details and outcome measurement. Seven studies investigated the baseline serum 25(OH)D level, and the majority of the participants were deficient. Four studies further evaluated the postintervention serum 25(OH)D level, which was markedly elevated compared with the normal concentration.

To evaluate pain, VAS or NRS was used. The VAS or NRS was assessed at baseline and at the end of each menstrual cycle throughout the study. Zarei S (2016) [19] used VAS to assess pain one month after the end of the study. Vit. D3 (cholecalciferol) was used in all studies as an intervention. Dosage widely varied among the studies from 5000 IU daily and 50,000 IU weekly to 300,000 IU monthly; the details are listed in Table 3. Calcium was added to the intervention and the control groups in the study by Zarei S (2016) [19]. Moreover, to be ethically compliant, painkillers were used in the intervention and the control groups among the studies conducted in the most recent 5-year period. The categories and amounts of pain relief medication were recorded. The regimens for the vit. D dose interval included daily use for 3 or 20 consecutive days in each cycle, regular weekly use, regular monthly use, or a single high dose at the beginning of the study. The duration of the studies ranged from two to four menstrual cycles.

### 3.5. Overall Effect Size (ES)

**Figure 2:**
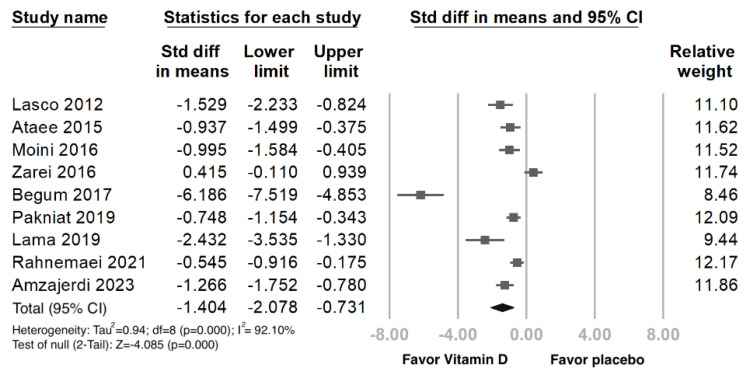
Forest plot intercept of the effect of vit. D interventions on pain relief in primary dysmenorrhea. Data were obtained from 9 published RCTs studies using the linear regression of the intervention effect Egger’s test. The pooled effect is represented using a black-colored solid diamond. The location of the diamond represents the estimated effect size and the width of the diamond reflects the precision of the estimate. The black-colored solid square marker size representing the effect size all have the same size and vary in size according to the weights assigned to the different studies. [Std diff, standardized difference.].

**Figure 3:**
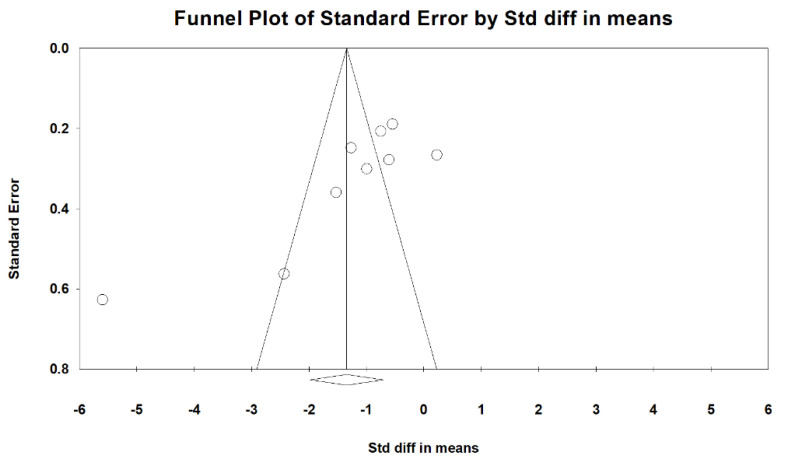
Contour-enhanced funnel: Egger’s plot of standard error (y-axis) by the standard difference in means (odds ratios x-axis). The vertical and diagonal lines represent the overall estimated effect size and its 95% confidence limits, respectively, based on the fixed-effect model. The shaded regions represent different significance levels for the effect size. [Std diff, standardized difference.].

Forest plot for the publication bias existed at the 5% significance level, the overall SMD of vit. D versus the placebo regarding primary dysmenorrhea was −1.404 (95% CI: −2.078 to −0.731) in the random-effects model, indicating a positive pain relief effect (Figure 2). We further performed Funnel: Egger’s test plot, the *p*-value was 0.025, and *I*^2^ (92.10%) revealed a high heterogeneity among the included studies (Figure 3). 

### 3.6. Subgroups Analysis

Results of the subgroup analysis based on the total vit. D dose, study time, and dose interval are listed in Table 4. In the total dose subgroup analysis, the average and total vit. D doses were over 50,000 IU and over 400,000 IU, respectively. These doses significantly reduced the pain reported by patients (SMD: −1.056; 95% CI: −1.619 to −0.493). Pain was also reduced with an average weekly consumption of vit. D of under 50,000 IU, (SMD −1.709; 95% CI: −2.947 to −0.472).

In a high-dose vit. D intervention (over 50,000 IU), pain was reduced regardless of intervention duration (over 70 days: SMD −2.856; 95% CI: −9.324 to 3.612; under 70 days SMD −1.122; 95% CI: −1.578 to −0.666).

The vit. D dose interval regimens included once, monthly, weekly, or daily, showing significant effects on the relief of pain when the weekly dose was over 50,000 IU. Therefore, we found that a high vit. D dosage could alleviate the pain associated with primary dysmenorrhea regardless of the dosing frequency and intervention period(s).

## 4. Discussion

This SRMA of nine RCTs focused on the effects of oral vit. D administration on primary dysmenorrhea, which demonstrated pain relief as a positive primary outcome in the intervention group. Pain levels were significantly lower when the average weekly dose of vit. D was over 50,000 IU. For those who were administered weekly doses of more than 50,000 IU, dysmenorrhea was relieved regardless of whether vit. D was taken for more than or less than 70 days and regardless of the dose interval.

A lower weekly dose also showed pain-relief effects, but the statistical significance of the dose could not be confirmed due to the wide weekly variation in the dose from 750 to 18,750 IU [19,22]. The majority of studies evaluated pain relief as an outcome in the second month/cycle [16,18,21]. A longer duration was used: 3 months in two studies [17,19] and 4 months in one [20]. As the duration of the menstrual cycle ranged from 21 to 35 days, as one of the inclusion criteria, our analysis of the study duration time was divided into two groups: over or under 70 days (two cycles). Various dose intervals for each regimen were found, including once in the study period (2/9) [20,23], monthly (3/9) [9,16,17], weekly (3/9) [18,21,22], and daily (1/9) [19]. Using the Endocrine Society’s Definition of vit. D sufficiency of 30 ng/mL, the level of vit. D insufficiency was 77% of the U.S. population according to the National Health and Nutrition Examination Survey (NHANES) 2001–2004 and 75.2% based on NHANES 2003–2006, which indicate that the vast majority of Americans can be considered as having an insufficient vit. D status [24].

Vit. D deficiency is related to menstrual dysfunction and pathogenesis [11]. A positive clinical correlation was found between vit. D deficiency (<12 ng/mL) and dysmenorrheal pain scores as well as other menstrual symptoms of depression, fatigue, and headache in a study of Turkish women (sample size 683) [25]. Conversely, a cross-sectional study conducted with 897 Iranian girls found no significant association between vit. D deficiency (<15 ng/mL) and menstrual symptoms [26]. These contradictory results may be explained by the differences in the cut-off value for defining vit. D deficiency.

Primary dysmenorrhea is related to both insufficient calcium consumption and, hence, low blood calcium and low vit. D levels [13]. One 25(OH)D intervention altered calcium homeostasis, which modulates the pathogenesis of primary dysmenorrhea [27]. As calcium influx plays a role in the modulation of smooth muscle contraction/relaxation, low blood calcium levels lead to the contraction of the uterine smooth muscle [28]. As it is hormone-like, vit. D has receptors throughout the body, with its action mediated by insulin-like growth factor-1. Moreover, 1-α hydroxylase, an enzyme, is responsible for vit. D metabolism, and it is expressed in the myometrium; this points to the critical role of vit. D in dysmenorrhea and its related symptoms [29,30]. Low levels of vit. D and calcium have an inverse relationship with the severity of primary dysmenorrhea. Notably, the 1000 mg calcium intervention reduced patient-reported pain, but there is a lack of evidence lacking with respect to the analgesic effect of the combination of vit. D and calcium.

Dysmenorrhea [31,32] is related to a key factor, prostaglandins (PGs), the level of which was found to be higher in the menstrual fluid of those with dysmenorrhea [33]. PGs are derived from the omega-6 fatty acids in the uterine cell membrane. After the onset of progesterone withdrawal before menstruation, these omega-6 fatty acids, particularly arachidonic acid, are released, triggering a cascade of PG formation in the uterus. These PGs are inflammatory, producing both uterine cramps and systemic symptoms of nausea, vomiting, and headache. In particular, PGF2α and PGE2 cause potent vasoconstriction (VC) and myometrial contractions, leading to uterine ischemia (the cause of primary dysmenorrhea) and, consequently, causing pain. As PGs are involved in both primary and secondary dysmenorrhea, the selective inhibition of PGE2 formation or the inhibition of its action via membrane receptor blockage is required to (i) inhibit adhesion, invasion, growth, and survival of endometriotic cells; (ii) decrease PGE2-induced inflammation and pain; (iii) decrease estrogen-dominant and progesterone-resistant states in the endometriotic lesions; and (iv) improve the endometrial microenvironment to provide fertilized ova implantation support and, hence, support pregnancy [34].

Vit. D can reduce PG production in the uterine tissue via several pathways. First, vit. D decreases PG receptor expression, which is followed by PG-mediated functional response inhibition. Second, vit. D speeds up PG degradation by enhancing 15-(dehydrogenase) enzyme activity. Finally, vit. D can decrease cyclooxygenase-2 (COX-2) expression in a manner similar to NSAIDs [35]. With the regulation of the upstream transcription factor nuclear factor kappa B cell (NF-κB), vit. D significantly reduces interleukin (IL)-1beta, IL-6, or the tumor necrosis factor-alpha (TNF-α)-induced inflammatory cytokine cascade (IL-8, PGE2, PGF2, and NF-κB activation [35]; AKT; mitogen activated protein kinase (MAPK); and Janus activated kinase/Signal transducer and activator of transcription 3 (JAK/STAT3)) [36]. Dysmenorrhea, which comprises related uterine hypercontraction, may contribute to myosin light-chain kinase activation, which is induced by calcium influx [28]. Vit. D alleviated L-type calcium-channel-related contraction and the calcium released in an ex vivo model [37], which indicates the potential mechanism of vit. D in dysmenorrhea. However, wide variations in the serum 25(OH)D levels after oral vit. D have been reported, starting from 40 IU per day and producing a 0.4 ng/mL serum level increase [38], 800 IU per day lasting for 8 weeks and producing a 0.5 ng/mL increase, and 1600 IU per day for 16 weeks and producing a 6 ng/mL increase in the serum 25(OH)D level [39], which may have led to the large heterogeneity in our study. The serum 25(OH)D level before and after oral vit. D intervention was tested in four out of nine RCTs, three of which reported 17.2, 45.7, and 50.1 ng/mL increases in serum vit. D levels for a total dose loading of 400,000 IU in 2 months [18,21,22]. The remaining study reported a 16.86 ng/mL increase after a 300,000 IU vit. D treatment over 4 months [40]. Larger increases in the serum 25(OH)D level were observed in populations where the starting level was low, with these increases slowing as the 25(OH)D concentration reached 40 ng/mL [15,41,42]. Thirteen case reports on vit. D toxicity from self-medication or the hypervitaminosis of vit. D deficiency were reviewed in 2018 [43]. Four of the thirteen cases reported oral vit. D overdose due to iatrogenic and labeling errors, including 970,000 IU in oral form for 1 month and 50,000 IU per day for 1 year, which resulted in serum vit. D concentrations ranging between 150 and 1220 ng/mL and serum calcium concentrations between 11.1 and 23.1 mg/dL. The reported symptoms of vit. D overdose-caused toxicity were vomiting, dehydration, pain, and a loss of appetite. None of the above overdoses and related adverse effects were found in the nine RCTs included in the current SRMA. The optimal 25(OH)D level was suggested to range from 30 to 150 ng/mL in the most recent reports [14,44]. However, some experts still chose 20 ng/mL as the cut-off value based on more evidence regarding bone health at this level [45]. With a weekly dose of 30,000 IU, 95% efficiency was achieved in increasing vitamin D levels to above 30 ng/mL. In contrast, with the administration of a low daily dose of 1000 IU, only 14% of participants were able to reach the desired vitamin D level of 30 ng/mL [46]. The recommended daily oral vit. D dose widely differs among guidelines: 600 IU vit. D per day was suggested by the Institute of Medicine [45]; 800–1000 IU per day was recommended for elderly patients by the International Osteoporosis Foundation [47]; and, finally, 1500–2000 IU of vit. D per day was recommended for adults by the Endocrine Society Clinical Practice Guideline [41].

### 4.1. Study Strengths

Despite primary dysmenorrhea being common, it is often undertreated. Effective and affordable potential therapies are available to improve the quality of life with decreased time lost from education or work (no-harm principle). As such, the strength of the current SRMA is that it addresses the benefits of recommending vit. D as a potential measure/treatment, apart from NSAIDs or OCPs.

### 4.2. Study Limitation

First, the diagnosis of primary dysmenorrhea relied on recording medical history without using ultrasound to exclude the anatomical lesions of secondary dysmenorrhea. Second, publication bias cannot be ignored, as studies with positive results are more frequently published, and those with negative results are hard to find. Both vit. D deficiency and dysmenorrhea are common in young women; thus, the intake of vit. D for primary dysmenorrhea relief could be one of the clinical options, especially in those experiencing side effects or contraindications to NSAIDs and/or OCPs use. Based on the current SRMA study, vit. D is effective for the relief of primary dysmenorrhea symptoms (positive significant results) using a weekly dose of up to 50,000 IU for 8 weeks. 

However, considering the possible occurrence of hypervitaminosis, monitoring the serum 25(OH)D level is suggested with the long-term use of vit. D.

## 5. Summary and Conclusions

In the current SRMA study, we pooled nine relevant RCTs. SRMA results showed that vit. D supplementation in women with primary dysmenorrhea and vit. D deficiency reduced the severity of dysmenorrhea-related pain. Second, vit. D supplementation decreased painkiller use and increased serum 25(OH)D levels. Third, a significant negative correlation between serum 25(OH)D levels and pain intensity, is a prominent finding. After vit. D supplementation intervention, we reported a significant difference in serum 25(OH)D levels between the patient and the negative control/placebo groups. 

According to the results of subgrouping analysis, a single dose of 300,000 IU vit. D administration 5 days before the menstrual cycle, as a loading phase, followed by a sustainability period of 60 to 120 days, could effectively relieve the degree of pain/distress in primary dysmenorrhea.

In the future, we prospect to explore the relationship between patients’ with primary menstrual pain and if they take other nutrients, such as the arginine amino acid, given involvement of arginine vasopressin receptor 1A, cysteine, and glycine, or glutamine amino acids, given glutamine involvement in glutathione S-transferase pi1 or theta1 genes https://www.ncbi.nlm.nih.gov/gene retrieved via gene sources such as the NCBI database (accessed on 5 March 2023). Micronutrients and trace elements such as vit. E can also be considered, per, their influences as antioxidants [48] or for affecting the endothelium, as well as vit. C with minerals such as zinc, magnesium, and selenium, required for glutathione function. A molecular epi/genetic mechanism should be applied to explore vit. D gene polymorphism [49] in order to provide a basis for nutrigenomics in a step toward precision nutrition for primary dysmenorrhea in the era of precision medicine.

## Figures and Tables

**Table 1:** Cochrane Collaboration’s tool for assessing the risk of bias (RoB) in the 9 studied RCTs to evaluate and analyze quality of the studies.

RCT Author Name Year [Ref.]	Bias				
	Selection	Performance	Detection	Attrition	Reporting
Lasco 2012 [16]	?	+	+	+	+
Ataee 2015 [17]	+	+	+	+	+
Moini 2016 [18]	?	+	+	+	+
Zarei 2016 [19]	+	+	+	+	+
Begum 2017 [20]	+	?	?	+	+
Pakniat 2019 [9]	+	+	−	+	+
Lama 2019 [21]	+	−	−	+	+
Rahnemaei 2021 [22]	+	+	+	+	+
Amzajerdi 2023 [23]	+	+	+	+	+

Risk of bias levels; low risk (+), high risk (−), and unknown (?).

**Table 2:** Demographics of RCTs included in the current SRMA.

RCT Author Name Year [Ref.]	Study Country	Sample Size	Age Range (Years)	Menstrual Cycle Intervals (Days)	Assessment Tool/Scale	Blindness Design
Lasco A (2012) [16]	Italy	40	18–40	21–35	VAS	Double
Ataee (2015) [17]	Iran	54	18–30	21–35	VAS	Double
Moini A (2016) [18]	Iran	50	18–30	NA	VAS	Double
Zarei S (2016) [19]	Iran	85	18–32	21–35	VAS	Double
Begum (2017) [20]	India	50	18–25	21–35	VAS	Single
Pakniat (2019) [9]	Iran	200	18–25	21–35	VAS	Single
Lama (2019) [21]	Saudi Arabia	22	13–40	NA	VAS	Open-label
Rahnemaei (2021) [22]	Iran	116	18–32	22–35	NRS	Double
Amzajerdi (2023) [23]	Iran	78	18–25	21–35	VAS	Double

Assessment scale, VAS/NRS; RCT blindness design, either single/double/open-label. [VAS, visual analogue scale; NRS, numerical rating scale.] NA, not applicable.

**Table 3:** Vit. D intervention details (level at start and end, dose, and dose interval) in the 9 RCT primary dysmenorrhea studies included in the current SRMA.

RCT Author Name Year [Ref.]	Study Duration (Months)	Serum 25(OH)D Level		Primary Pain Score		Dosage Frequency	Dose Interval	Total Dose	Weekly Dose	Note/ Remarks	Significant Results for
		Vit. D	Control	Vit. D	Control	IU	(Time)	IU	IU		
Lasco A (2012) [16]	0	27.2 ± 7.5	29.9 ± 7.6	5.85 ± 2.0	5.6 ± 1.9	300,000 single	month	600,000	75,000	Control/placebo pill Intervention time: 5 days before next period	D
	1	NA	NA	3.7 ± 1.34	5.4 ± 1.8						
	2	NA	NA	3.5 ± 1.3	5.7 ± 1.6						
Ataee (2015) [17]	0	7.3 ± 3.6	6.3 ± 2.8	7.13 ± 1.85	7.38 ± 1.56	300,000 single	month	900,000	75,000	Control/placebo pill Intervention time: 5 days before next period	D
	1	NA	NA	5.30 ± 2.24	5.21 ± 2.35						
	2	NA	NA	3.97 ± 1.90	5.24 ± 2.27						
	3	NA	NA	3.77 ± 1.77	5.55 ± 2.02						
Moini A (2016) [18]	0	9.7 ± 5.09	11.5 ± 3.7	8.50	9.60	50,000 weekly	week	400,000	50,000	Control/placebo pill	D
	2	55.4 ± 6.02	13.57 ± 4	2.50	7.60						
Zarei S (2016) [19]	0	NA	NA	6.20 ± 1.60	6.30 ± 1.80	5000	day	300,000	25,000	Control/placebo + 1000 mg Ca Intervention time: 20 days /menstruation period	Ca > Ca + D >placebo
	1			5.40 ± 2.30	4.7 ± 2.30						
	2			4.70 ± 2.20	4.2 ± 2.20						
	3			4.60 ± 2.60	3.6 ± 2.20						
	4			5.00 ± 2.60	3.9 ± 2.50						
Begum (2017) [20]	0	17.8 ± 10.1	19.10	8.76 ± 0.97	8.80 ± 0.95	300,000 single	once	300,000	18,750	Control/placebo pill	D
	2	NA	NA	2.72 ± 1.17	8.20 ± 0.74						
	4	34.7 ± 8.1	19.34	3.56 ± 0.76	8.20 ± 0.74						
Pakniat (2019) [9]	0	NA	NA	7.01 ± 0.11	7.24 ± 0.56	3000	month	6000	750	Vit D: 3000 IU + 500 mg mefenamic acid	D
	2	NA	NA	5.20 ± 1.34	6.00 ± 0.70					Control/placebo + 500 mg mefenamic	
Lama (2019) [21]	0	30.1 ± 13.4	19.5 ± 5.5	7.8 ± 1	6.9 ± 1.2	50,000	week	400,000	50,000	Vit D: 50,000 IU + NSAID Control: NSAID	D
	2	80.2 ± 14.3	19.70 ± 5.6	3.6 ± 1.2	6.4 ± 1.1						
Rahnemaei (2021) [22]	0	20 ± 6	19.5 ± 5.5	7.0 ± 1.7	6.6 ± 1.5	50,000	week	400,000	50,000	Vit D: 50,000 IU +NSAID or acetaminophen	D
	2	37.2 ± 9.4	19.7 ± 5.6	5.6 ± 1.7	6.5 ± 1.6						
Amzajerdi (2023) [23]	0	5.10 ± 3.31	6.6 ± 5.63	6.71 ± 2.25	6.64 ± 2.46	300,000 single	once	300,000	18,750	Control/placebo pill	D
	1	30.63 ± 5.43	9.73 ± 4.72	5.33 ± 2.39	6.53 ± 2.30						
	2			3.92 ± 2.36	6.79 ± 2.17						

Data are mean ± SD. Mefenamic acid is an NSAID; pain score scales 0–10, evaluated by VAS or NRS. [NSAID, nonsteroidal anti-inflammatory drug; NA, not available; VAS, visual analogue scale; NRS, numerical rating scale; D, vit. D.]

**Table 4:** Subgroup analysis of standardized mean differences (SMDs) based on total vit. D intervention dose, RCT duration, and vit. D dose interval for different average weekly dose(s).

Subgroup	SMD	95% CI
Total dose		
Average weekly ≥ 50,000 IU		
a. Total dose ≥ 400,000	−1.056 *	−1.619 to −0.493
b. Total dose < 400,000	NA	NA
Heterogeneity (Tau^2^ = 0.225, df (Q) = 3 (*p* = 0.013), *I*^2^ = 72.287%)		
Average weekly < 50,000 IU		
a. Total dose ≥ 400,000	NA	NA
b. Total dose < 400,000	−1.709 *	−2.947 to −0.472
Heterogeneity (Tau^2^ = 1.851, df (Q) = 4 (*p* = 0.000), *I*^2^ = 95.569%)		
Study duration		
Average weekly ≥ 50,000 IU		
a. Study duration ≥ 70 days	−0.937 *	−1.499 to −0.375
Heterogeneity (Tau^2^ = 0.000, df (Q) = 0 (*p* = 1.000), *I*^2^ = 0.000%)		
b. Study duration < 70 days	−1.171 *	−2.007 to −0.334
Heterogeneity (Tau^2^ = 0.422, df (Q) = 2 (*p* = 0.005), *I*^2^ = 81.236%)		
Average weekly < 50,000 IU		
a. Study duration ≥ 70 days	−2.856	−9.324 to 3.612
Heterogeneity (Tau^2^ = 21.518, df (Q) = 1 (*p* = 0.000), *I*^2^ = 98.773%)		
b. Study duration < 70 days	−1.122 *	−1.578 to −0.666
Heterogeneity (Tau^2^ = 0.092, df (Q) = 2 (*p* = 0.097), *I*^2^ = 57.066%)		
Dose interval		
Average weekly ≥ 50,000 IU		
a. Monthly or once	−0.937 *	−1.499 to −0.375
Heterogeneity (Tau^2^ = 0.000, df (Q) = 0 (*p* = 1.000), *I*^2^ = 0.000%)		
b. Weekly or daily	−1.171 *	−2.007 to −0.334
Heterogeneity (Tau^2^ = 0.422, df (Q) = 2 (*p* = 0.005), *I*^2^ = 81.236%)		
Average weekly < 50,000 IU		
a. Monthly or once	−2.258 *	−3.617 to −0.899
Heterogeneity (Tau^2^ = 1.762, df (Q) = 3 (*p* = 0.000), *I*^2^ = 94.925%)		
b. Weekly or daily	0.415	−0.110 to 0.939
Heterogeneity (Tau^2^ = 0.000, df (Q) = 0 (*p* = 1.000), *I*^2^ = 0.000%)		

* Significant difference at *p* < 0.05. [SMD, standardized mean differences; CI, confidence interval; NA, not applicable.]

## Data Availability

Data are contained within the article.

## Abbreviations

C	Comparator
CI	Confidence interval
CKAP2L	Cytoskeleton-associated protein 2-like
COX-2	Cyclooxygenase 2
GWAS	Genome-wide association studies
I	Intervention
IL	Interleukin
JAK	Janus activated kinase
KEGG	Kyoto Encyclopedia of Genes and Genomes
MAPK	mitogen activated protein kinase
MMPs	Matrix metalloproteinases
NF-κB	Nuclear factor kappa B cell
NRS	Numerical rating scale
NSAIDs	Non-steroidal anti-inflammatory drugs
O	Outcome
OCPs	Oral contraceptive pills
P	Population
PCOS	Polycystic ovary syndrome
PGs	Prostaglandins
RCTs	Randomized clinical trials
ROB	Risk-of-bias
RoB 2	Cochrane risk of bias
SMD	Standardized mean difference
SRMA	Systemic review meta-analysis
STAT3	Signal transducer and activator of transcription 3
TNF-α	Tumor necrosis factor-alpha
V.C	Vasoconstriction
VAS	Visual analogue scale
VDR	Vitamin D receptor
VEGF	Vascular endothelial growth factor
Vit. D2	Ergocalciferol
Vit. D3	Cholecalciferol
Vit.D	Vitamin D
